# Identification of a Population of Epidermal Squamous Cell Carcinoma Cells with Enhanced Potential for Tumor Formation

**DOI:** 10.1371/journal.pone.0084324

**Published:** 2013-12-20

**Authors:** Gautam Adhikary, Dan Grun, Candace Kerr, Sivaprakasam Balasubramanian, Ellen A. Rorke, Mohan Vemuri, Shayne Boucher, Jackie R. Bickenbach, Thomas Hornyak, Wen Xu, Matthew L. Fisher, Richard L. Eckert

**Affiliations:** 1 Department of Biochemistry and Molecular Biology, The University of Maryland School of Medicine, Baltimore, Maryland, United States of America; 2 Department of Dermatology, The University of Maryland School of Medicine, Baltimore, Maryland, United States of America; 3 Department of Reproductive Biology, The University of Maryland School of Medicine, Baltimore, Maryland, United States of America; 4 Department of Microbiology and Immunology, The University of Maryland School of Medicine, Baltimore, Maryland, United States of America; 5 The Marlene and Stewart Greenebaum Cancer Center, The University of Maryland School of Medicine, Baltimore, Maryland, United States of America; 6 Life Technologies, Inc. Stem Cell Technologies, Frederick, Maryland, United States of America; 7 Department of Anatomy and Cell Biology, University of Iowa Carver College of Medicine, Iowa City, Iowa, United States of America; University of Texas MD Anderson Cancer Center, United States of America

## Abstract

Epidermal squamous cell carcinoma is among the most common cancers in humans. These tumors are comprised of phenotypically diverse populations of cells that display varying potential for proliferation and differentiation. An important goal is identifying cells from this population that drive tumor formation. To enrich for tumor-forming cells, cancer cells were grown as spheroids in non-attached conditions. We show that spheroid-selected cells form faster growing and larger tumors in immune-compromised mice as compared to non-selected cells. Moreover, spheroid-selected cells gave rise to tumors following injection of as few as one hundred cells, suggesting these cells have enhanced tumor-forming potential. Cells isolated from spheroid-selected tumors retain an enhanced ability to grow as spheroids when grown in non-attached culture conditions. Thus, these tumor-forming cells retain their phenotype following *in vivo* passage as tumors. Detailed analysis reveals that spheroid-selected cultures are highly enriched for expression of epidermal stem cell and embryonic stem cell markers, including aldehyde dehydrogenase 1, keratin 15, CD200, keratin 19, Oct4, Bmi-1, Ezh2 and trimethylated histone H3. These studies indicate that a subpopulation of cells that possess stem cell-like properties and express stem cell markers can be derived from human epidermal cancer cells and that these cells display enhanced ability to drive tumor formation.

## Introduction

Epidermal squamous cell carcinoma ranks among the most common forms of human cancer. Moreover, due to environmental irritants and exposure to UV irradiation, the incidence is increasing [1]. Thus, skin cancer is an important health concern. In early disease, the cancerous lesion can be removed by surgical excision. However, the high frequency of skin cancer means that treatment is expensive and advanced disease is life-threatening and disfiguring.

 It is widely appreciated that large numbers of tumor cells (millions) must be injected into immune-suppressed mice to produce palpable tumors. It has been suggested that may be because only a small percentage of cells, within the larger population, is capable of forming tumors. Recent evidence in several systems suggest that tumors contain a small subpopulation of cells, called cancer stem cells (CSC), which exhibit self-renewal capacity, proliferate infrequently, and are responsible for tumor maintenance and metastasis [2]. Moreover, it has been proposed that these “slow cycling” cells are not impacted by anti-cancer agents that kill rapidly growing tumor cells [3]. Since the cancer stem cells are thought to give rise to other cells in the tumor, eliminating the stem cell population may be necessary to halt tumor formation [3].

 Substantial progress has been made in identifying human cancer stem cell markers. In breast cancer, the stem cell population is CD44^+^/CD24^-^ [4], and CD133 marks cancer stem cells in brain tumors, colorectal carcinoma, and pancreatic carcinoma [5–8]. In head and neck squamous cell carcinoma, a CD44^+^ population of cells possesses the properties of CSC [9], and aldehyde dehydrogenase 1 (ALDH1) activity has also been reported to identify cancer stem cells in a host of cancer types [10–13]. The human epidermis contains multiple stem cell populations [2], including the CD200^+^/K15^+^/K19^+^ hair bulge stem cells [14] and the α6^+^/β1^+^/CD71^-^ interfollicular stem cells [15,16]. CD133 has also been reported to identify human skin cancer stem cells [17–19].

 Cancer cells with enhanced tumor forming potential can be selected by cell sorting [4] or by growth as spheroids [20,21]. In the present study, we utilize human epidermal stem cell markers and non-attached growth conditions to isolate and characterize epidermal squamous cell carcinoma cells with enhanced potential to form tumors. These cells were enriched by selection in non-attached culture conditions. The selected cells form fast growing tumors in immune-compromised mice at lower densities as compared to non-selected cells, and express many proteins that mark epidermal stem cells. These cells may represent a population of squamous cell carcinoma cancer stem cells.

## Results

### Characterization of skin cancer stem cells

**Figure 1:**
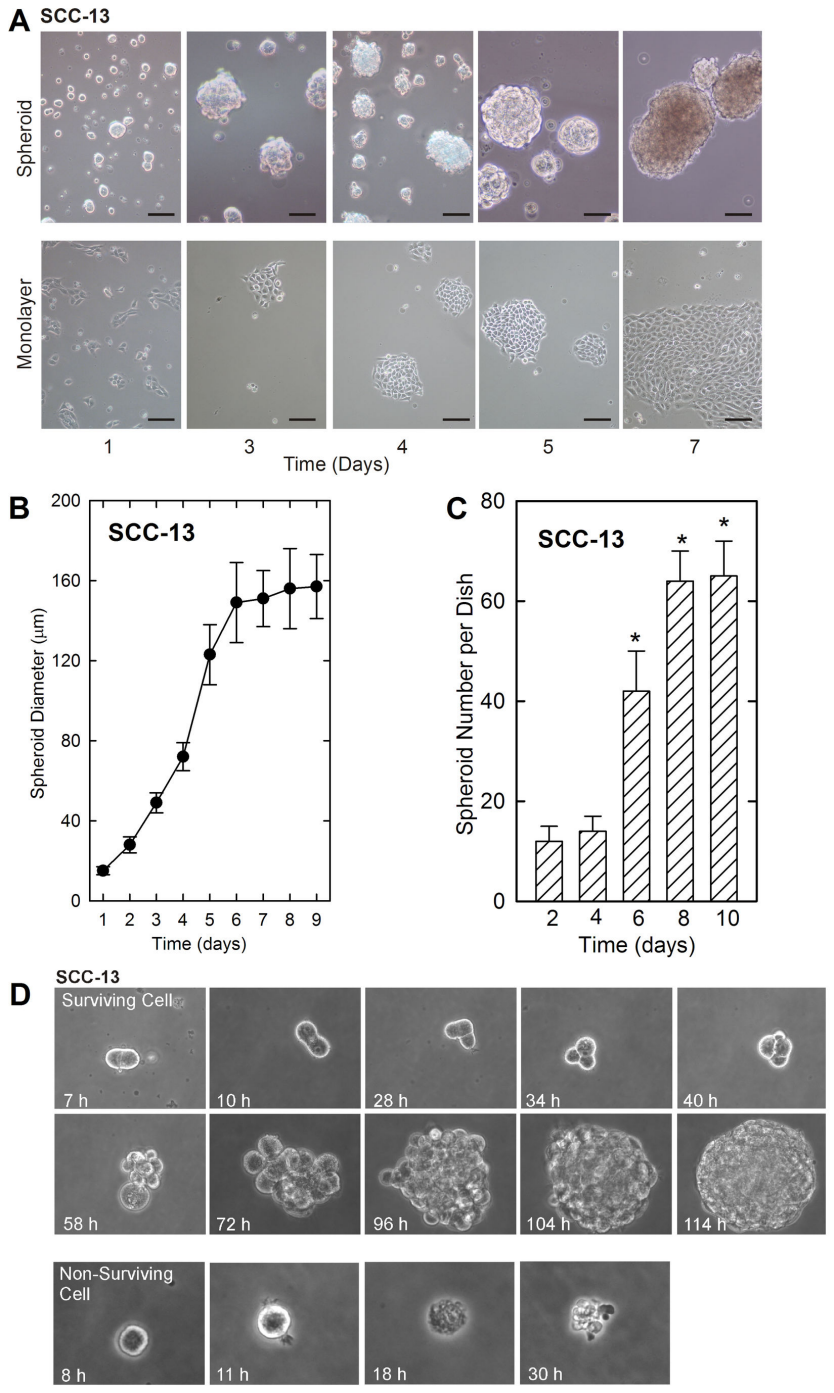
A subpopulation of SCC-13 cells grow as spheroids. **A** SCC-13 monolayer cultures, maintained in growth medium, were harvested and plated at 40,000 cells per 9.5 cm^2^ in poly-HEMA coated dishes or in standard tissue culture plates and grown in spheroid medium. Monolayer and P1 spheroid cultures were monitored for growth. The bars = 50 μm. **B** SCC-13 spheroid growth rate. SCC-13 cells were plated on poly-HEMA coated dishes and the diameter of P1 spheroids was monitored for 0 - 9 d. The values are mean + SEM, n = 3. **C** Spheroids are formed from a subset of SCC-13 cells. SCC-13 cells (40,000 single cells) were plated on poly-HEMA coated dishes and the total number of P1 spheroids was monitored for 0 - 10 d. Care was taken to assure that spheroid formation was not due to cell aggregation. A small percentage of cells (0.15%) are able to form spheroids. The values are mean + SEM, n = 4. The asterisks indicate a significant increase in spheroid number as compared to the day 2 data point (p < 0.05). **D** Selection of spheroid-forming cells. SCC-13 monolayer cultures were dissociated and plated into 96 well non-attachment plates at one cell per well. At the indicated times after plating, the cells were photographed. The upper panel shows on of the 0.15% of cells that survived and formed a spheroid. The bottom panel shows one of the non-surviving cells undergoing cell death.

Growth as non-attached multicellular spheroids can be used to select cancer cells with enhanced tumor forming potential [22,23]. We applied this method to determine whether tumor forming cells can be isolated by growing human epidermis-derived SCC-13 cells as spheroids. Figure **1A** compares the growth of SCC-13 cells in non-attached and monolayer conditions. Forty-thousand cells were seeded and colony expansion was monitored for 7 days. Monolayer growth produces colonies that expand with a typical cobblestone appearance. In contrast, the cells in non-attached culture form multicellular spheroids that grow in size until they plateau as colonies with a 150 - 160 μm diameter (Figure **1B**). Counting of the number of spheroids formed from these cultures indicate that seeding forty-thousand cells results in formation of sixty spheroids (Figure **1C**). This indicates that only 0.15% of the cells in these cultures are able to grow as spheroids. To assess whether this was a selection process, we plated single SCC-13 cells into 96 well low-attachment plates and monitored cell survival. This clonal survival assay confirmed that 0.15% of the cells survive this process. Figure **1D** shows examples of these two cell fates as monitored at the indicated times after cell plating. The fact cell death occurs within hours after plating, strongly suggests the culture conditions provide selection pressure to derive spheroid-forming cells. Thus, these finding are consistent with a small population of cells giving rise to these structures [22].

**Figure 2:**
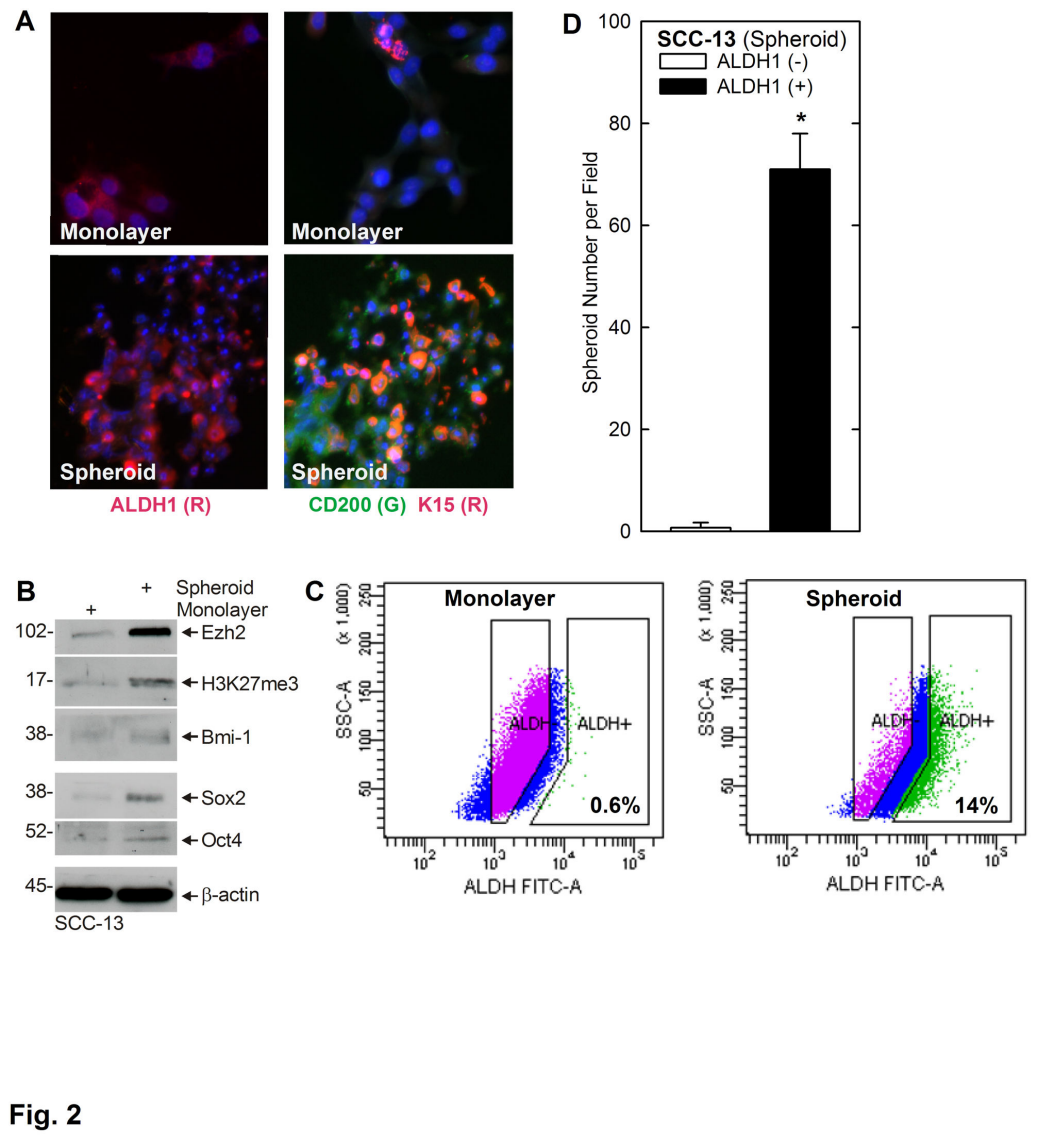
SCC-13 spheroid cultures express stem cell-related marker proteins. SCC-13 cells, maintained in growth medium, were harvested and grown as P1 spheroids on non-attached (spheroid) or attached (monolayer) conditions. **A** Spheroid cultures express stem cell marker proteins. After 10 d, P1 spheroids and monolayer cultures were harvested, and the cells were permitted to attach on glass coverslips and stained with anti-ALDH1, anti-CD200 or anti-K5. No signal was observed in absence of primary antibody (not shown). **B** Spheroid cultures express stem cell marker proteins. Ten day spheroid and monolayer cultures were harvested for preparation of total extract, and the indicated epitopes were detected by immunoblot. **C** P1 spheroids are enriched in ALDH^+^ cells. P1 monolayer and spheroid cultures were grown for 10 d, harvested as single cell suspensions, incubated with ALDH1 substrate, and ALDH1 positive and negative cells were quantified by flow cytometry. The boxes indicate the sorting windows for cells considered ALDH+ and ALDH-. **D** ALDH1^+^ cells form spheroids. ALDH1(+) and ALDH1(-) cells, isolated from 10 d P1 spheroid cultures by cell sorting, were plated as a single cell suspension at 40,000 cells per 9.5 cm^2^ well on non-attachment dishes for a 10 d spheroid formation assay. At 10 d the number of spheroids were counted. The values are mean + SEM, n = 3. The asterisk indicates that spheroid formation by ALDH1(+) cells is significant greater as compared to ALDH1(-) cells (p < 0.05).

 Cancer stem cells express a characteristic set of stem cell markers that vary in a tumor type-specific manner, but typically include proteins that mark stem cells from the parent tissue. We used this property to characterize the spheroid cells. The human epidermis includes the CD200^+^/K15^+^/K19^+^ hair bulge stem cells [14] and the α6^+^/β1^+^/CD71^-^ interfollicular stem cells [2,15,16]. To characterize the SCC-13 cell spheroids, we stained to detect CD200 and K15, and compared expression in monolayer versus spheroid cultures. Figure **2A** shows that the spheroids are enriched for CD200 and K15 expression as compared to monolayer cultures. Cell extracts were obtained for immunoblot detection of additional makers. Figure **2B** shows that spheroid cultures are also enriched for expression of Sox 2, Oct 4, Bmi-1 and Ezh2. Ezh2 is a histone methyltransferase that trimethylates histone H3 on lysine 27 (H3K27me3) [24–26]. Moreover, the spheroid cell-associated increase in Ezh2 level is associated with enhanced Ezh2 activity, as evidenced by increased H3K27me3 formation (Figure **2B**). These studies indicate that spheroid cultures derived from SCC-13 cells express markers that are characteristic of stem cells.

 Stem cells are frequently positive for aldehyde dehydrogenase 1 (ALDH1) [10,27–29]. Immunostaining of spheroid cultures with anti-ALDH1 shows a specific increase in ALDH1-positive cells (Figure **2A**). In addition, we harvested monolayer and first passage (P1) spheroids and sorted for ALDH1+ cells. This analysis reveals that 0.6% of the cells in monolayer culture versus 14% of spheroid-derived cells are ALDH1^+^ (Figure **2C**). We further assessed whether ALDH1-positive phenotype is associated with enhanced ability to form spheroids. For this purpose, P1 spheroid cultures were sorted to isolate ALDH1^-^ and ALDH1^+^ cells, and the cells were replated at equal density on cell non-adherent dishes and monitored for spheroid formation. Spheroid formation is 60-fold more efficient for ALDH1^+^ as compared to ALDH1^-^ cells (Figure **2D**).

**Figure 3:**
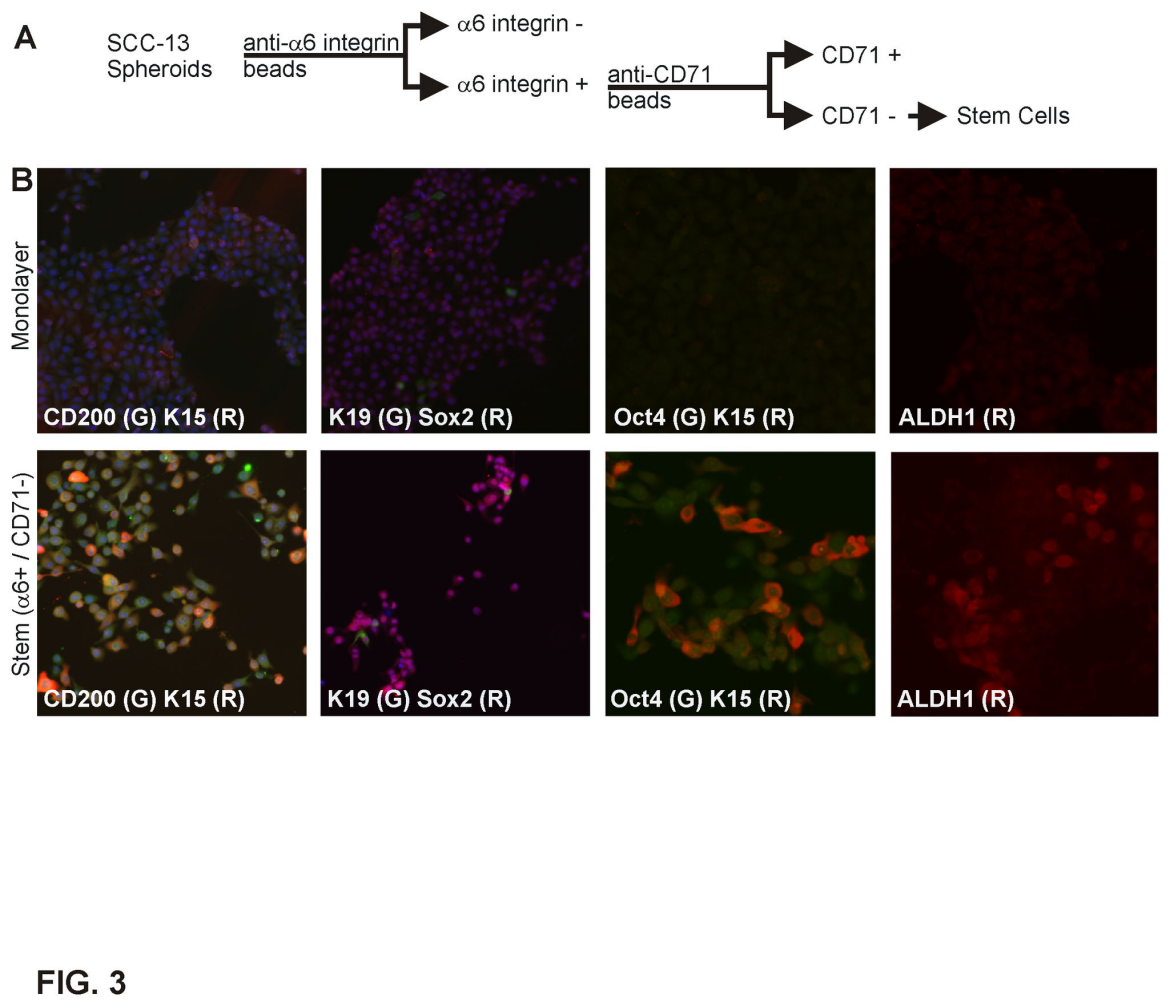
Stem cell marker expression in SCC-13 α6-integrin^+^/CD71^-^ cells. **A** Isolation of α6-integrin^+^/CD71^-^ cells. Dissociated cells from 10 d spheroids were incubated with anti-α6 integrin beads to select for anti-α6 integrin positive cells which were then incubated with anti-CD71 beads to remove CD71^+^ cells to yield the α6-integrin^+^/CD71^-^ cells. **B** Spheroid-derived α6-integrin^+^/CD71^-^ cells express epidermis-relevant stem cell markers. α6-integrin^+^/CD71^-^ cells were isolated using anti-α6-integrin and anti-CD71 beads as outlined above. These cells were then stained with anti-CD200 (green), anti-K15 (red), anti-K19 (green), anti-Sox2 (red), anti-Oct4 (green) and anti-ALDH1 (red) and visualized by confocal microscopy. Similar results were observed in each of three experiments.

 As mentioned above, normal interfollicular epidermal stem cells are α6-integrin-positive and CD71-negative [15]. We hypothesized that cancer cells may express these stem cell markers. To test this, we used magnetic bead-conjugated antibodies to purify α6^+^/CD71^-^ cells from SCC-13 monolayer and spheroid cultures and then assayed for expression of other stem cell markers (Figure **3A**) [30]. The selected cells displayed elevated levels of CD200, K15, Sox 2 and Oct 4 (Figure **3B**). Taken together, these findings suggest that growth in non-attached conditions selects for a population of SCC-13 cells that are enriched in expression of stem cell markers [2].

### Spheroid-derived cells display enhanced ability to form tumors

**Figure 4:**
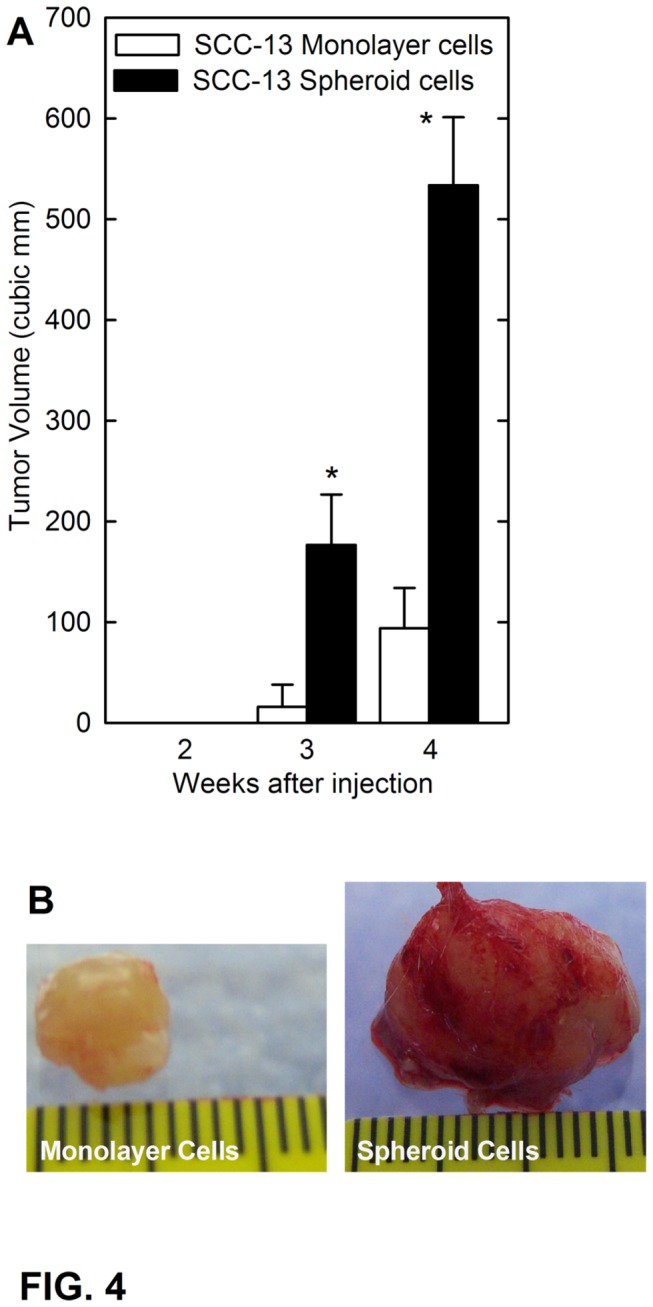
SCC-13 spheroid-selected cells display enhanced tumor formation. **A** SCC-13 spheroid- or monolayer-derived cells were injected at 100,000 cells per site in NSG mice and tumor growth was monitored. Tumors volume was calculated as 4/3π x (diameter/2)^3^ [64]. The values are mean + SEM for 8 individual tumors. Asterisks indicate significant differences in tumor size between the spheroid and the monolayer groups at each time point (p < 0.005, n = 8). **B** Representative spheroid- and monolayer-derived tumors were harvested on week four and photographed. The spheroid-derived tumors are larger and red (vascularized) in appearance.

The cancer stem cell model predicts that a small population of stem cells is responsible for tumor formation and that enriched populations of such cells will preferentially initiate tumor formation [4,17,18,31]. We therefore assessed whether the spheroid-derived cells display enhanced tumor formation compared to cells derived from monolayer culture. One hundred thousand monolayer or spheroid-derived cells were injected subcutaneously in NSG mice, and tumor growth was monitored over a period of four weeks. These studies show that spheroid cell-derived tumors grow much larger than tumors derived from monolayer cells (Figure **4A**). Moreover, visual inspection reveals that the spheroid cell-derived tumors are better vascularized than the monolayer cell-derived tumors (Figure **4B**).

**Figure 5:**
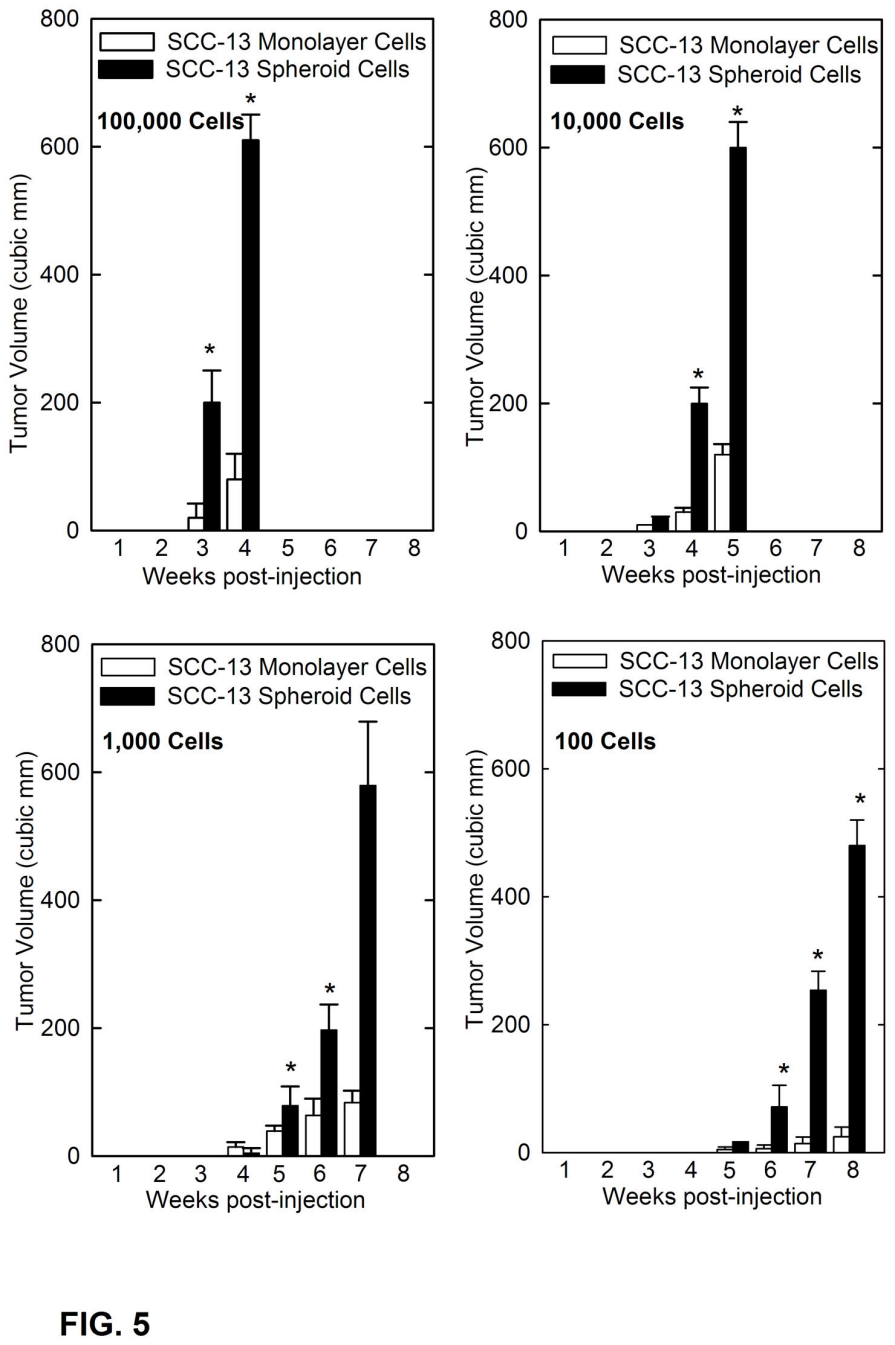
Spheroid-selected cells form tumors at lower injection densities. SCC-13 spheroid- and monolayer-derived cells were injected at 100 to 100,000 cells per each of two or four sites in NSG mice (three to ten mice injected per cell number). Tumor formation was monitored by palpation and tumor size values are in cubic millimeters. The values are mean + SEM. Asterisks indicate significant differences in tumor size between the spheroid group and the monolayer group at each time point (p < 0.005).

 We next assessed the impact of a reduced injection density on tumor formation. Although it is difficult to demonstrate in practice, in theory, a single stem cell should be able to give rise to a tumor [4]. To assess the potential to form tumors at reduced injection density, spheroid- and monolayer-derived SCC-13 cells were injected into NSG mice at 10 to 100,000 cells per site, and tumor formation was monitored over a period of eight weeks (Figure **5**). The first observation is that time to onset of tumor formation is reduced with reduced cell injection density. This is best appreciated by noting the week at which tumors reached a size of 200 cubic millimeters. This time increases from three weeks following injection of 100,000 spheroid-derived cells to seven weeks following injection of 100 spheroid-derived cells. The second observation is that spheroid cell-derived tumors expand much more efficiently, compared to monolayer cell-derived tumors, at all cell injection levels. This difference is particularly evident when only 100 cells are injected (Figure **5**). We show that only 0.15% of SCC-13 cells derived from monolayer culture are able to form spheroids (Figure **1C**). If these cells correspond to the tumor forming cells, it is not surprising that tumor formation is minimal when 100 monolayer culture-derived cells are assayed for tumor formation, and no tumors are formed following injection of ten monolayer-derived cells. In contrast, injection of 100 spheroid-derived cells formed large tumors that reach a size of 500 cubic millimeters in eight weeks. These findings suggest that spheroid-derived cells more efficiently form tumors.

### Spheroid cell-derived tumors retain phenotype

**Figure 6:**
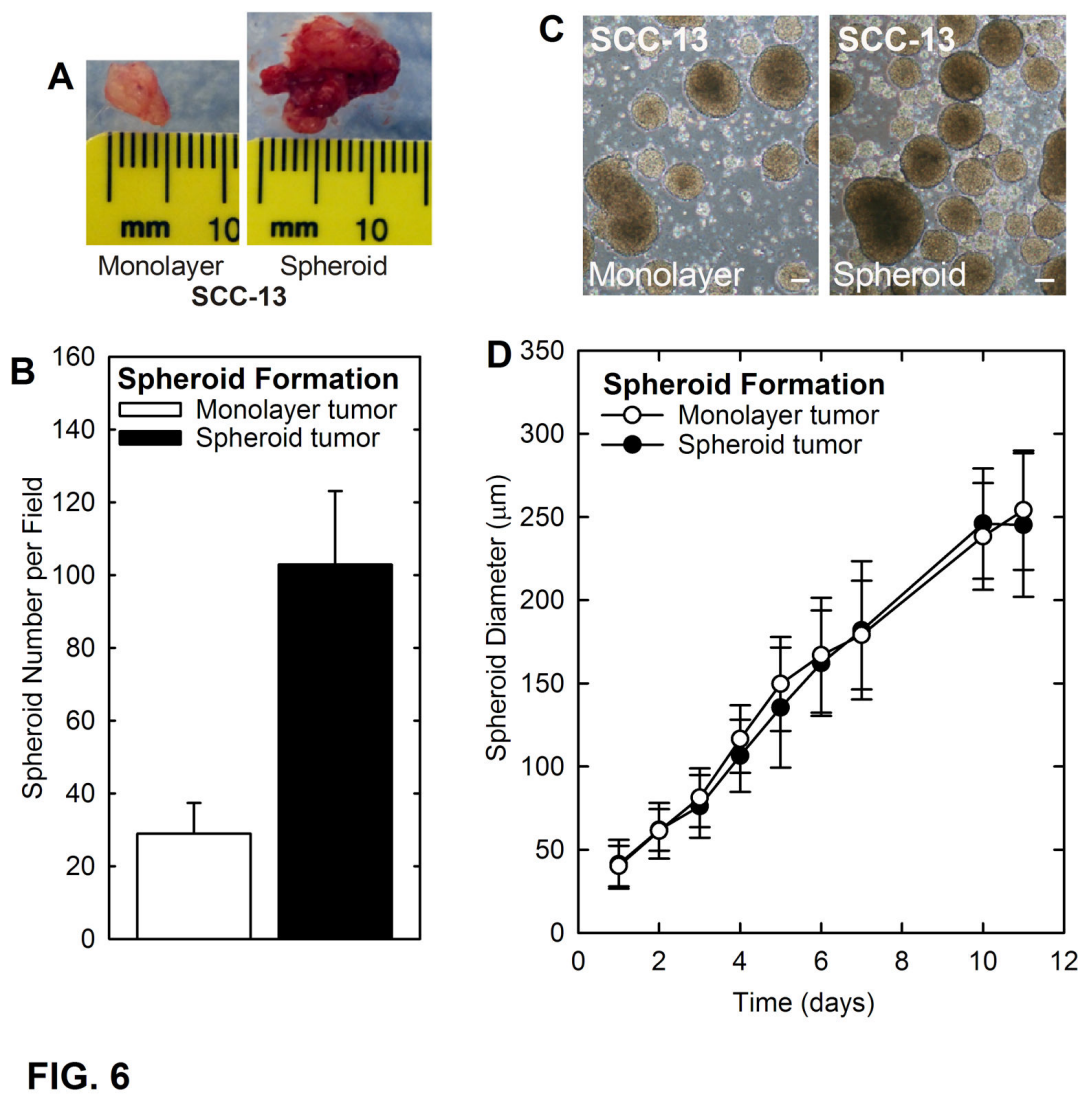
Spheroid-selected skin tumor cells retain properties during *in* *vivo* growth. **A** Morphology of tumors derived following injection of 100,000 spheroid-selected and non-selected (monolayer) cells after growth for four weeks. **B/C** Cells derived from spheroid-selected and non-selected tumors were dissociated and ability to form spheroids in culture was monitored. Spheroid formation is significantly greater for tumors formed from spheroid-selected cells. The values are mean + SEM, n = 4 independent tumors per group (p < 0.005). The bars = 100 μm. The photographs were taken after 10 d of spheroid growth. **D** Spheroid growth rate is identical for tumor cells derived from spheroid-selected and monolayer cells. Forty thousand cells, harvested from the tumors derived from spheroid-selected and non-selected cells, were plated in spheroid selection medium to monitor the rate of spheroid growth. Values are the mean + SEM (n = 4 independent tumors per group). No significant difference is observed in spheroid growth rate for cells derived from monolayer and spheroid tumors.

Tumor cells differ in growth rate, ability to invade surrounding tissue and ability to support neovascularization, and selection of increasingly invasive and malignant tumor cells is a process that occurs during cancer progression. The above studies indicate that spheroid growth conditions select epidermal SCC cells with enhanced ability to form tumors. A key question is whether these cells retain spheroid formation ability after growth as tumors. To assess this, one hundred thousand spheroid-derived and monolayer-derived cells were injected for tumor formation. Four week tumors (Figure **6A**) were enzymatically dissociated and single cell suspensions were assayed for ability to form spheroids. The plot in Figure **6B** shows that spheroid tumor-derived cells form four-fold more spheroids than monolayer tumor-derived cells. This suggests that the spheroid cells maintain the spheroid formation phenotype during *in vivo* passage. Figure **6C** shows that the spheroids are morphologically identical to those before passage. We also compared the spheroid growth rate of cells derived from monolayer and spheroid tumors. This analysis shows that spheroids derived from both tumor types grow at an identical rate (Figure **6D**). These findings suggest that spheroid-forming cells are present in both cell populations, but are enriched in spheroid-selected cultures.

### Spheroid enrichment identifies A431 tumor-forming cells

**Figure 7:**
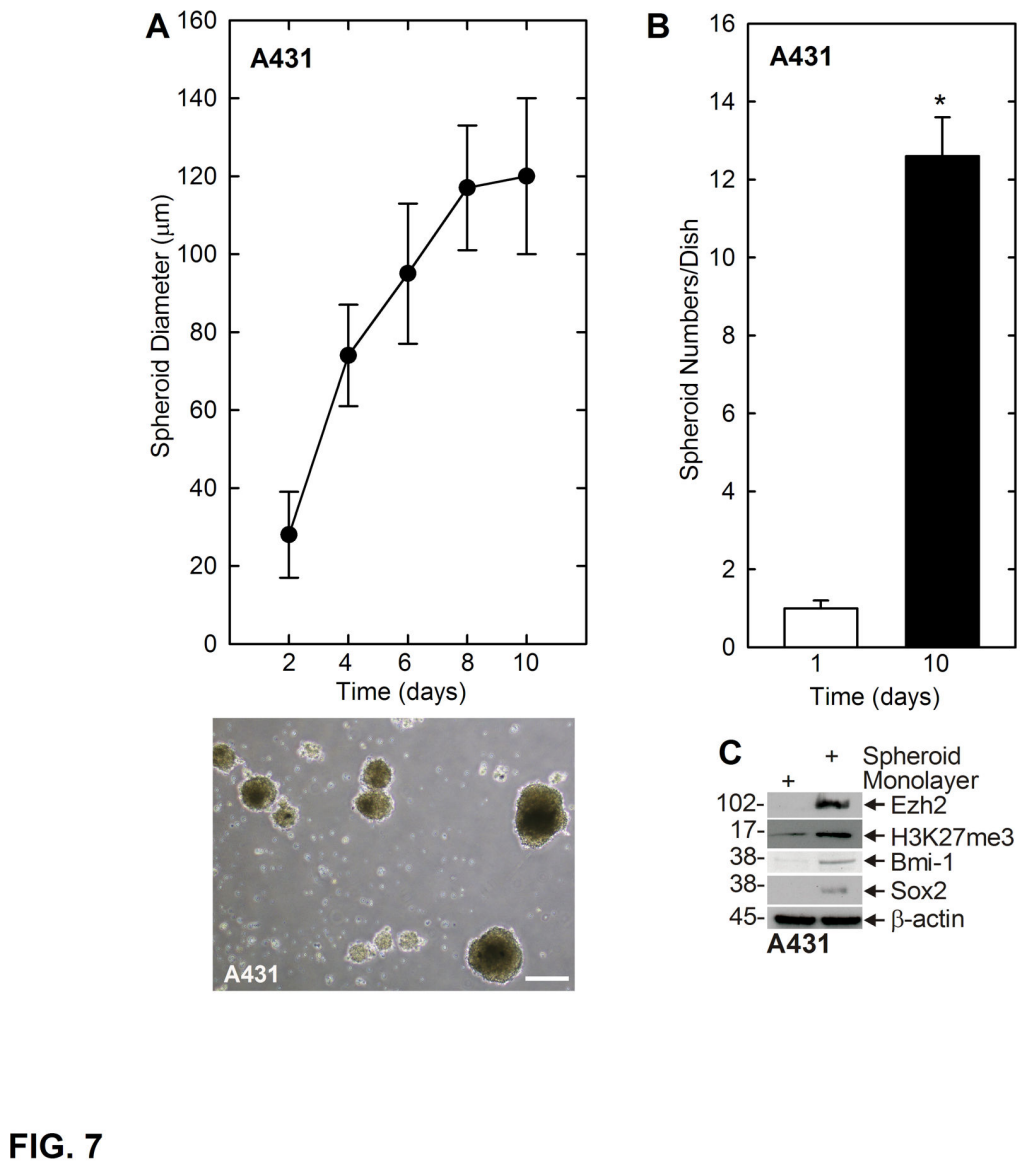
A431 skin cancer cells contain a population of stem cell marker-positive cells with enhanced ability to form tumors. **A** A431 cells form spheroids. A431 cells were plated at 40,000 cells per 9.5 cm^2^ well in spheroid-selection medium and the rate of spheroid formation and morphology were recorded. The bottom panel is an image of P1 spheroids after 10 d of growth. **B** A subpopulation of A431 cells form spheroids. A431 cells (40,000) were plated in spheroid growth conditions and spheroid number was monitored on days 1 and 10. Among the 40,000 cells plated in this assay, only 0.03% survive and form spheroids. The asterisk indicates a statistically significant increase in spheroid number at day 10 compared to day 1 (p < 0.005, n = 3) **D** Spheroid-selected A431 cells express stem cell markers. Ten day spheroid and monolayer A431 cultures were harvested and extracts were assayed for expression of the indicated stem cell markers by immunoblot.

**Figure 8:**
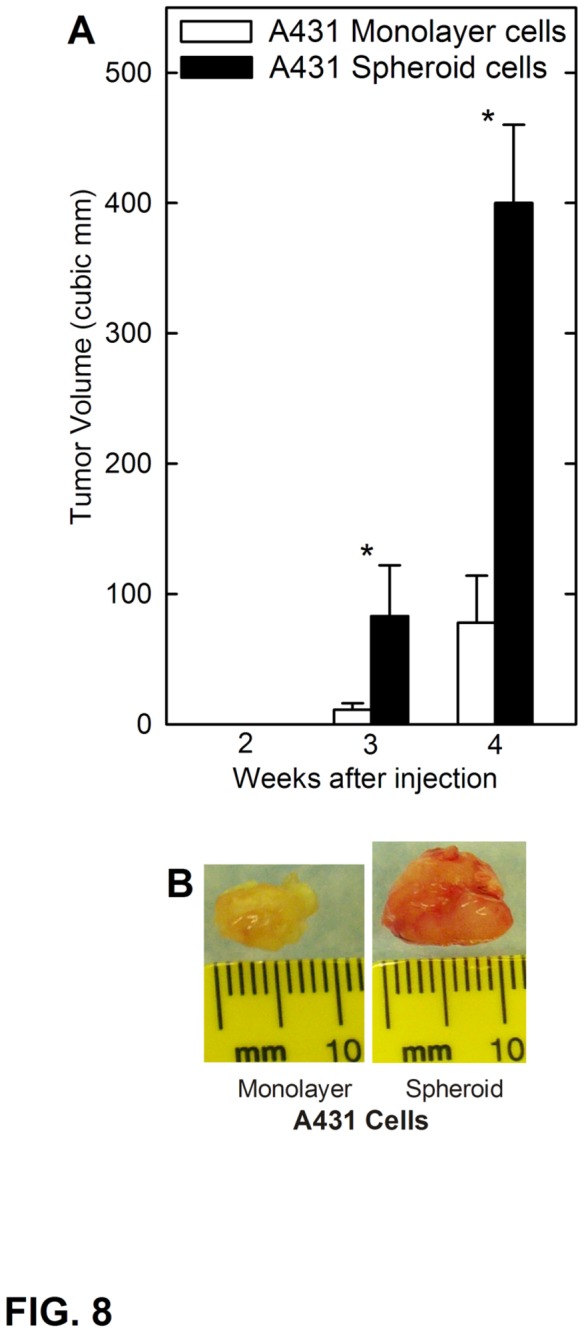
Spheroid-selected A431 cells display enhanced tumor formation. **A** Spheroid- or monolayer-derived A431 cells were injected at 500,000 cells per site into each of the four sites in NSG mice and tumor growth was monitored. Tumor volume was calculated as 4/3π x (diameter/2)^3^ [64]. The values are mean + SEM for six individual tumors. Asterisks indicate significant differences in tumor size between the spheroid and the monolayer groups at each time point (p < 0.005, n = 8). B Spheroid- and monolayer-derived A431 cell tumors were harvested on week four and photographed. The spheroid-derived tumors are larger and appear vascularized (red).

The above studies focused on SCC-13 cells. To confirm these findings, we assessed whether a population of spheroid-forming cells could be derived from the A431 epidermal squamous cell carcinoma cell line. Monolayer-derived A431 cells were harvested and 40,000 cells were seeded in non-attachment plates and maintained under spheroid-growth conditions identical to that used for the SCC-13 cells. A431 spheroids, display a typical morphology, and expanded in size to reach a plateau diameter of 120 μm at ten weeks (Figure **7A**). Plating of 40,000 cells results in formation of twelve spheroids (Figure **7B**), indicating that only 0.03% of A431 cells are ability to grow as spheroids. To characterize the A431 cell spheroids, we prepared extracts and stained to detect a selected set of stem cell markers. Figure **7C** shows that A431 spheroid-derived cells are enriched for expression of Ezh2, H3K27me3, Bmi-1 and Sox2. These studies indicate that spheroid cultures derived from A431 cells express markers that are characteristic of stem cells. We also assessed the ability of monolayer and spheroid culture-derived cells to produce tumors in mice. In these experiments, 0.5 million cells were injected and tumor formation was monitored as a function of time. Figure **8A** shows that spheroid-derived A431 cells form larger tumors than monolayer-derived cells. Figure **8B** shows that these tumors are also highly vascularized as compared as compared to monolayer-derived cultures. Taken together, these studies indicate that A431 cells contain a tumor-forming cell subpopulation that is similar to that observed in SCC-13 cells.

## Discussion

 In this report we describe a highly tumorigenic subpopulation of cells derived from cultures of epidermal squamous cell carcinoma cells. To our knowledge this is the first characterization of tumor forming cells derived from human cancer cells lines derived from epidermis. We identified a subpopulation (0. 15%) of cells that survive when selected for growth as spheroids. These cells self-renew and can be carried for multiple passages in spheroid-selection conditions, a characteristic of cancer stem cells [32]. Marker analysis reveals that these cells are highly enriched for markers that identify stem cells in normal human epidermis *in vivo*.

### Tumor-forming cells express epidermal and embryonic stem cell markers

Human epidermis contains multiple stem cell populations [2]. Major populations responsible for keratinocytes renewal include the CD200^+^/K15^+^/K19^+^ hair bulge stem cells [14] and the α6^+^/β1^+^/CD71^-^ interfollicular stem cells [15,16]. CD133 also identifies human skin cancer stem cells [17–19]. We anticipated that tumor-forming cells derived from a skin cancer cell population would selectively express a subset of markers which may signify a single subpopulation of epidermal stem cells, either hair bulge or interfollicular, as the source of the cells. However, analysis reveals that the cells are positive for both bulge (CD100, K15 and K19) and interfollicular epidermal stem cell markers (α6 integrin). To confirm this finding, we used magnetic beads to select α6^+^/CD71^-^ cells and then assayed for expression of markers of hair bulge stem cells. The selected cells were positive for CD200 and K19, which mark hair follicle cells. Thus, our studies suggest that cancer forming cells express markers from multiple epidermal stem cell populations. The prevailing idea, derived from mouse studies, is that the cells that accumulate carcinogen and give rise to tumors reside in the hair bulge [33]. However, the precise relationship among stem cell populations in epidermis is not well understood, and recent studies suggest that the stem cells which give reside in the interfollicular epidermis and hair follicle may be related. Thus, it may not a surprising that squamous cell carcinoma stem cells express markers characteristic of stem cells derived from multiple niches.

 In addition, these cells express elevated levels of embryonic stem cell markers, including Sox2, Oct4 and the polycomb group proteins (Ezh2, Bmi-1). All of these proteins are important in maintenance of stem cell survival. Oct4 and Sox2 are overexpressed in some cancer stem cell types [34]. Examples include human oral, prostate and breast carcinoma [34–37] and are sometime selectively elevated in advanced cancer [34,36,38]. The observation that SCC-13 cell derived spheroids are enriched in Oct4 and Sox2 suggest that they share properties with embryonic stem cells and may reflect the fact that they are transformed [34,39].

 Polycomb group (PcG) proteins repress gene expression by modifying chromatin structure [40,41]. PcG proteins function as two complexes. The PRC2 complex includes Ezh2, Suz12, eed, and RBAP48. The catalytic subunit is Ezh2, a methyltransferase that trimethylates lysine 27 of histone H3 (H3K27me3) [41]. The other subunits are required for optimal Ezh2 activity [42]. The PRC1 complex includes Ring1B, Bmi-1, PH1 and CBX [41]. The catalytic subunit, Ring1B, ubiquitinates H2A-K119 and is optimally active in association with Bmi-1 [43]. An important role of the CBX protein is interaction with H3K27me3 to anchor the PRC1 complex to chromatin [24]. Once positioned in chromatin, Ring1B ubiquitinylates H2A-K119 which leads to a closed chromatin state. PcG protein expression is increased in cancer cells and tumors and this is linked to reduced tumor suppressor expression and increased cancer cell proliferation and survival [24,40]. We have recently showed that Ezh2 level is increased in epidermal squamous cell carcinoma and is required for skin cancer cell survival [44–48], a finding that is consistent with results in other tumor cell systems [49–53]. Our present studies extend these findings and show that Ezh2 and Bmi-1 levels are substantially enriched in SCC tumor forming stem cells as compared to non-stem cells. Moreover, Ezh2 expression is associated with increased H3K27me3 formation. H3K27me3 formation is a chromatin mark that is associated with silencing of tumor suppressor gene expression [54]. These findings have fundamental implications for the development of new cancer therapeutics. Tumor response is typically defined by tumor shrinkage. However, cancer stem cells may be inherently resistant to traditional therapeutic agents that target proliferating cancer cells. This may be why tumor regression often does not translate to a significant increase in patient survival [55]. To achieve a significant clinical outcome, it may be necessary to develop specific therapeutics that target cancer stem cells. It is interesting to speculate that some of the stem cell survival proteins we have localized in skin cancer stem cells, including Oct4, Sox2, Ezh2 and Bmi-1, may provide such targets. For example, Ezh2 inhibitors are already in development [56,57].

### Spheroid-selected cells display enhanced tumor formation

The tumor stem cell hypothesis predicts that only a small subset of tumor cells is able to efficiently drive tumor formation [32,34]. In solid tumors, it is known that a large number of cells must be injected to achieve tumor formation [4]. This is thought to be because only a specific subpopulation of cells has the ability to form a tumor, and that these are present as a small fraction of the total cell number. This implies that enrichment of these cells will lead to enhanced tumor formation. Indeed, we observe that the population of cells that is enriched by growth in spheroid conditions has enhanced ability to form tumors. In fact, xenograft tumor formation studies, using spheroid-selected SCC-13 cells, indicate that injection of as few as 100 cells results in robust formation of tumors that grow to a size of 500 mm^3^. It is interesting that these cells are highly enriched for expression of stem cell markers. This compares to formation of relatively few small (10 - 30 mm^3^) tumors for non-selected cells that lack stem cell marker expression.

 In addition, visual analysis indicates that spheroid-enriched cells give rise to blood vessel-enriched tumors that are multi-lobed and highly integrated into the surrounding tissue. This suggests that individual cells give rise to various lobes of each tumor. This is in contrast to the small, less vascularized and compact tumors observed for non-selected cell populations. The enhanced vascularization is an interesting property. Recent studies indicate that keratinocytes express the VEGF receptor and produce VEGF, and that VEGF production is enriched in skin cancer stem cells [58,59]. Thus, it may not be surprising that stem cell-derived tumors display enhanced angiogenesis. These tumor-forming cells may produce and release VEGF and other pro-vascularization agents that stimulate tumor stem cell survival and also cause endothelial cells to migrate to the tumor. An additional interesting feature is that the spheroid-selected cells are able to form spheroids after passage (as tumors) *in vivo*. This suggests that passage through the animal tumor environment does not “erase” the spheroid-formation phenotype, and suggests that these cells retain stem cell-like properties.

 Finally, we assessed whether another skin cancer cell line, A431 cells, displays similar properties. We show that a small subpopulation (0.03%) of these cells survive in culture to form spheroids. This compares favorably to the 0.15% value observed in SCC-13 cells. Moreover, spheroid-selected A431 cells express increased levels of stem cell markers, including Bmi-1, Sox2, Ezh2 and H3K27me3, and spheroid-selected A431 cells are highly efficient tumor forming cells. Moreover, the tumors are highly vascularized as compared to non-selected A431 cells. These finding suggest that we have identified an important subpopulation of cells in SCC-13 and A431 cells that possess the ability to form tumors. Several recent reports also describe a subpopulation of A431 cells that from spheroids and have enhanced tumor formation potential. These studies demonstrate, among other features, that these cells are able to undergo epithelial-mesenchymal transition [60–62]. These studies are consistent with an enhanced ability of these A431-derived stem cells to form tumors [61,62].

## Materials and Methods

### Antibodies and reagents

DMEM (11960-077), sodium pyruvate, (11360-070), L-Glutamine (25030-164), penicillin-streptomycin solution (15140-122), 0.25% trypsin-EDTA (25200-056) were purchased from Gibco (Grand Island, NY). Heat-inactivated fetal calf serum (FCS, F4135) was obtained from Sigma. Antibodies for Ezh2 (612667) and Oct4 (611203) were obtained from BD transduction laboratories (San Jose, CA). Anti-H3K27me3 (07-449) was from EMD Millipore (Bedford, MA). Antibodies of Sox2 (ab15830-100), CD200 (ab23552), Bmi-1 (ab14389) and ALDH1 (ab23375) were obtained from Abcam. Anti-α6-integrin (SC-0374057) was from Santa Cruz Biotechnology (Santa Cruz, CA). Anti-K15 (10137-1-AP) was obtained from ProteinTech (Chicago, IL), and anti-K19 (MMS-158S) was from Covance (Dedham, MA). β-Actin (A5441) antibody was purchased from Sigma (St. Louis, MO). Anti-mouse IgM magnetic microbeads (#130-047-301) and anti-CD71 (transferrin receptor) (#130-046-201) magnetic microbeads were obtained from Miltenyi Biotech (Cambridge, MA). Alexa Fluor 594 goat anti-rat IgG (A11007), Alexa Fluor 488 goat anti-mouse IgG (A21121) and Alexa Fluor 594 goat anti-rabbit IgG (A11012) secondary antibodies were obtained from Invitrogen and used at 1:500 dilution. Peroxidase-conjugated anti-mouse IgG (NXA931) and anti-rabbit IgG (NA934V) were obtained from GE Healthcare (Buckinghamshire, UK) and used at a 1:5000 dilution. Statistical comparisons were made using the t-test.

### Cell spheroid selection conditions

SCC-13 and A431 cells were obtained from Dr. James Rheinwald [63] and American Type Culture Collection, respectively. These cells were maintained in growth medium (DMEM, Invitrogen, Frederick, MD, supplemented with 4.5 mg/ml D-glucose, 200 mM L-glutamine, 100 μg/ml sodium pyruvate, 100 U/ml penicillin, 100 U/ml streptomycin and 5% fetal calf serum). For spheroid formation assay, 80% confluent cultures were harvested with trypsin and gently pipetted to form a single cell suspension. Trypsin was inactivated by addition of serum-containing medium and the cells were collected by centrifugation at 2,000 rpm for 5 min. The cells were resuspended in spheroid medium which is DMEM/F12 (1:1) (DMT-10-090-CV, Mediatech Inc (Manassa, VA) containing 2% B27 serum-free supplement (17504-044, Invitrogen, Frederick, MD), 20 ng/ml EGF (E4269, Sigma, St. Louis), 0.4% bovine serum albumin (B4287, Sigma) and 4 μg/ml insulin (Sigma, St. Louis, MO, #19278) and plated at 40,000 cells per 9.5 cm^2^ well in six well ultra-low attachment cluster dishes (#3471, Corning, Tewksbury, MA) or 1.2% poly-HEMA coated dishes. Poly-HEMA was prepared by suspending 1.2 g poly-HEMA (P3932, Sigma, 2-hydroxyethyl methacrylate) per 100 ml 95% ethanol and heating at 65 °C with mixing. Dishes were coated by addition of 2.5 ml of poly-HEMA stock per 9.5 cm^2^ dish in a sterile hood. The ethanol was permitted to evaporate overnight and the dishes were sterilized by 1 h UV irradiation. Parallel control cultures were plated in spheroid medium and grown as attached monolayers on conventional plastic dishes. At the time of plating the suspension contained 100% single cells. The number of passages wherein the cells were grown as spheroids is indicated by passage number, P1, P2, etc.

### Immunoblot

For immunoblot analysis, equivalent amounts of protein were electrophoresed on denaturing and reducing 8% polyacrylamide gels and transferred to nitrocellulose membrane. The membrane was blocked by 5% nonfat dry milk and then incubated with the appropriate primary (1:1000) and secondary antibody (1:5000). Secondary antibody binding was visualized using chemiluminescence detection technology.

### Immunostaining

SCC-13 cells were grown as monolayer or non-attached (spheroid) cell cultures. For immunostaining, cells were harvested, suspended in spheroid medium, and plated in 35 mm glass bottom wells (MatTek Corporation, P35G-1.0-14-C). This permits the cells to attach to a substrate for the purposes of immunostaining. After 16 h, the cells were fixed with 4% paraformaldehyde at room temperature for 15 min, washed three times with 1 x phosphate-buffered saline (PBS), incubated with 0.2% Triton X-100 for 10 min, washed three times with PBS, and blocked for 1 h with PBS containing 7.5% fetal calf serum. Primary antibodies were added and the slides incubated overnight at 4 °C. Cells were then washed three times with PBS, and incubated 1 h with appropriate Alexa Flour fluorescence probe-conjugated secondary antibody. After additional washing, the cells were stained with DAPI for 10 min. Confocal images were obtained using an Olympus IX81 spinning disk confocal microscope. For staining of microbead-separated cells, the cells were suspended in spheroid medium and permitted to attach to 35 mm glass bottom dishes from MatTek (P35G-1.0-14-C) for 16 h prior to fixation and processing.

### Tumor xenograft growth assays

Monolayer or spheroid-derived cancer cells were prepared as a single cell suspension by trypsin treatment, resuspended in phosphate buffered saline containing 30% Matrigel and 100 μl containing 100 to 100,000 cells was injected subcutaneously at four sites in the ventral flanks of NOD *scid* IL2 receptor gamma chain knockout mice (NSG mice) using a 26.5 gauge needle. Three to ten mice were used per data point (two or four tumors per mouse), depending upon the number of cells/site that were injected. Tumor growth was monitored by measuring tumor diameter and calculating tumor volume using the formula, volume = 4/3π x (diameter/2)^3^ [64]. Mice were euthanized by injection of 250 μl of a 2.5% stock of Avertin per mouse followed by cervical dislocation of the neck. Tumor samples were harvested to prepare extracts for immunoblot and sections for immunostaining. These experiments were reviewed and approved by the University of Maryland-Baltimore Institutional Animal Care and Use Committee. 
